# Ketogenic diets, physical activity and body composition: a review

**DOI:** 10.1017/S0007114521002609

**Published:** 2022-06-28

**Authors:** Damoon Ashtary-Larky, Reza Bagheri, Hoda Bavi, Julien S. Baker, Tatiana Moro, Laura Mancin, Antonio Paoli

**Affiliations:** 1 Nutrition and Metabolic Diseases Research Center, Ahvaz Jundishapur University of Medical Sciences, Ahvaz, Iran; 2 Department of Exercise Physiology, University of Isfahan, Isfahan, Iran; 3 Centre for Health and Exercise Science Research, Department of Sport, Physical Education and Health, Hong Kong Baptist University, Kowloon Tong, Hong Kong; 4 Department of Biomedical Sciences, University of Padua, Padua, Italy; 5 Human Inspired Technology Research Center, University of Padua, Padua, Italy; 6 Research Center for High Performance Sport, UCAM, Catholic University of Murcia, Murcia, Spain

**Keywords:** Body composition, Fat-free mass, Fat mass, Resistance training, Endurance training, Obesity, Ketogenic diet

## Abstract

Obesity remains a serious relevant public health concern throughout the world despite related countermeasures being well understood (i.e. mainly physical activity and an adjusted diet). Among different nutritional approaches, there is a growing interest in ketogenic diets (KD) to manipulate body mass (BM) and to enhance fat mass loss. KD reduce the daily amount of carbohydrate intake drastically. This results in increased fatty acid utilisation, leading to an increase in blood ketone bodies (acetoacetate, 3-*β*-hydroxybutyrate and acetone) and therefore metabolic ketosis. For many years, nutritional intervention studies have focused on reducing dietary fat with little or conflicting positive results over the long term. Moreover, current nutritional guidelines for athletes propose carbohydrate-based diets to augment muscular adaptations. This review discusses the physiological basis of KD and their effects on BM reduction and body composition improvements in sedentary individuals combined with different types of exercise (resistance training or endurance training) in individuals with obesity and athletes. Ultimately, we discuss the strengths and the weaknesses of these nutritional interventions together with precautionary measures that should be observed in both individuals with obesity and athletic populations. A literature search from 1921 to April 2021 using Medline, Google Scholar, PubMed, Web of Science, Scopus and Sportdiscus Databases was used to identify relevant studies. In summary, based on the current evidence, KD are an efficient method to reduce BM and body fat in both individuals with obesity and athletes. However, these positive impacts are mainly because of the appetite suppressive effects of KD, which can decrease daily energy intake. Therefore, KD do not have any superior benefits to non-KD in BM and body fat loss in individuals with obesity and athletic populations in an isoenergetic situation. In sedentary individuals with obesity, it seems that fat-free mass (FFM) changes appear to be as great, if not greater, than decreases following a low-fat diet. In terms of lean mass, it seems that following a KD can cause FFM loss in resistance-trained individuals. In contrast, the FFM-preserving effects of KD are more efficient in endurance-trained compared with resistance-trained individuals.

Obesity remains a significant public health concern throughout the world. According to the latest data from the WHO, the prevalence of obesity is increasing, with 13 % of adults worldwide classified as obese and 39 % classified as overweight^(1)^. Associated co-morbidities such as CVD, type 2 diabetes mellitus and various types of cancers are expected to rise dramatically in conjunction with the global obesity epidemic^(2–4)^. While increasing efforts continue to combat this disease, body mass (BM) loss strategies remain a complex and challenging dilemma for health care practitioners and individuals with obesity. Various dietary strategies have long been proposed for BM loss. One popular dietary strategy is classifying a diet based on macronutrient intake, including fat, protein and carbohydrate. Based on dietary carbohydrate intakes, diets can be classified as very-low-carbohydrate ketogenic diet (KD) (<5 % carbohydrates or <50 g/d), very-low-carbohydrate diet (LCD) (<10 % carbohydrates), LCD (<25 % carbohydrates or <130 g/d), moderate-carbohydrate diet (25–44 %) and high-carbohydrate diet (45 % or greater)^(5–7)^.

Nowadays, a low-carbohydrate approach is a popular strategy for decreasing BM and fat mass (FM). Based on the previously mentioned classifications, a KD is a very LCD, high in fat, with variation in protein intake but may be classified as moderate or high^(8)^. This macronutrient distribution leads to an increase in the production of ketone bodies (KB) and consequently to physiological ketosis (i.e. blood KB concentrations between 1 and 4 mm and blood potential of hydrogen (pH) of ≈ 7·4)^(9)^.

The literature outlines that carbohydrate-restricted diets (LCD and KD) are increasingly used to manage various health conditions, including neurological disorders, obesity, dyslipidaemia, hypertension, diabetes, the metabolic syndrome and various cancers^(6,10)^. As a result, carbohydrate-restricted diets have gained substantial popularity. In the USA, The Health Information National Trends Survey of 5586 participants reported among respondents who were aware of carbohydrate-restricted diets that approximately 17 % had tried LCD during the last year and one-third of respondents who were aware of LCD confirmed that they are employing a healthy strategy to control BM^(11)^. In the UK, media reports suggest that 7 % of men and 10 % of women are experimenting with carbohydrate-restricted diets^(12)^ and similar population values are reported from Finland^(13)^.

KD may act as a viable strategy for BM loss, particularly in the short term; however, BM loss may be accompanied by a loss of lean mass. Due to the importance of BM and the relevance of properly maintaining body composition^(14)^, the efficacy of KD on BM and body composition is an intriguing area of experimental research^(15,16)^. A focus on body composition during BM loss is critical to monitor changes in FM while maintaining or even improving lean mass^(17)^. A KD-derived BM loss programme is acknowledged as an efficient intervention within the first few weeks of implementation^(18)^. However, it has been suggested that a significant amount of BM loss includes reductions in lean mass and FM with changes in body fluid status^(19)^.

Nevertheless, the evidence for body composition alterations during a KD is inconclusive. Therefore, we aim to review the current evidence regarding the impact of various KD on body composition, with a focus on changes in body fat (FM or body fat percentage (BFP)) and lean mass. We will also critique the methodologies used to evaluate changes in body composition in athletes and individuals who are overweight and obese.

## Literature search

A literature search from 1921 to April 2021 using Medline, Google Scholar, PubMed, Web of Science, Scopus and Sportdiscus databases was used to identify relevant studies. The following keywords, alone or in conjunction, were used to find relevant articles: ‘ketogenic diet’, ‘very-low-carbohydrate high-fat diet’, ‘very-low-carbohydrate diet’, ‘carbohydrate-restricted diet’, ‘VLCD’, ‘body composition’, ‘weight’, ‘fat mass’, ‘fat-free mass’, ‘lean body mass’, ‘muscle mass’, ‘keto-adaptation’, ‘athletes’, ‘obesity’, ‘obese’, ‘overweight’, ‘resistance training’, ‘strength training’, ‘endurance training’, ‘aerobic training’, ‘high intensity interval training’ and ‘HIIT’. All eligible studies were in English. For this review, the inclusion criteria focused on using KD alone or in combination with exercise on BM loss and changes in lean mass and body fat. All studies had to provide a detailed explanation of their KD protocol. Studies included both males and females. As described in the following paragraph, a KD can vary slightly in the composition of the macronutrients and thus can be classified differently. In this review, we have considered only studies that used diets with <50 g/d and/or <5 % of carbohydrates and we will refer generically to a KD or very LCD throughout the manuscript.

## History and definition of ketogenic diet

The KD has been studied periodically for more than 100 years^(20,21)^. However, over the past 30 years, a growing body of research has suggested that a link exists between the process of KD adaptation and a broad range of health benefits^(20)^. Dr. Russel Wilder first used this type of diet to treat epilepsy in 1921^(22)^ and described the term ‘ketogenic diet.’ Because of Wilder’s observed beneficial results, the KD assumed a place in medical nutrition as a therapeutic diet for paediatric epilepsy and was widely used until its popularity declined as antiepileptic agents were introduced^(23,24)^. The classic KD is a type of very-low-carbohydrate and high-fat diet that concurrently restricts energy content. Typically, carbohydrate intake is reduced to <30 g/d; however, studies show that this number is not necessarily consistent to induce ketosis and fluctuates between 20 and 50 g/d^(9,24,25)^.

This diet serves to mimic a fasting state by shifting the utilisation of fats as a primary fuel source via the catabolism of fatty acids in the liver. KB are produced by the liver^(26)^. Nutritional ketosis is a clinically benign and physiological^(27)^ metabolic state that should not be confused with a pathological state of ketoacidosis, a hazardous complication of conditions including diabetes mellitus or alcoholism^(28)^. Ketosis in individuals typically leads to maximum blood KB concentrations of 4–5 mm, whereas concentrations in ketoacidosis often exceed ten times these values^(29)^.

## Types of ketogenic diets

There are several versions of the KD. However, we considered only the following types of KD, which are more readily available in the scientific literature. In addition to the explanations, Table 1 summarises the information.

**Table 1.** Types of ketogenic diets (KD)

Type of KD	Abbreviation	Macronutrient distribution	Characteristics
Classic ketogenic diet	Classic KD	CHO: 4% Fat: 90% Pro: 6%	Created for the management of childhood seizures
The Modified Atkins Diet	MAD	CHO: 5% (10 g for children and 20 g for adult) Fat: 65% Pro: 30%	No energy restriction
Very low-energy ketogenic diet	VLCKD	CHO: 13% (<30 g/d) Fat: 44% Pro: 43% (1.2–1.5 g/kg)	Mimics fasting through a noticeable restriction of daily carbohydrate and energy (<800 kcal) intake
Ketogenic Mediterranean diet/Modified Mediterranean Ketogenic diet	KMD or MMKD	CHO: <30/50 g/d Fat: 45–50% Pro: 30–35%	Emphasising on the intake of lean meats, fish, olive oil, walnuts and green vegetables and, in some protocols, the addition of herbal extracts

CHO, carbohydrate.

### Classic ketogenic diet

Historically, classic KD was proposed by Dr. Wilder in a series of patients with epilepsy in the Mayo Clinic^(22)^. The classic therapeutic KD (fat = 90 %, protein = 6 %, carbohydrate = 4 %), initially created to manage childhood seizures, has a 4:1 ratio of grams of fat:grams carbohydrate plus protein^(30,31)^.

### The modified Atkins diet

Modified Atkins diet limits the amount of carbohydrates consumed to 10–20 g/d (10 g for children and 20 g for adults), which was introduced as an alternative to the classic KD in 2003^(32)^. Modified Atkins diet does not restrict energy content, fluid or protein and allows a greater portion of carbohydrate and protein intake than the classic KD^(33)^ (e.g. fat = 65 %, protein = 30 %, carbohydrate = 5 %)^(32)^.

### Very low-energy ketogenic diet

Very low-energy ketogenic diet is a nutritional intervention that mimics fasting through a noticeable restriction of daily carbohydrate intake, usually lower than 30 g/d (≃13 % of daily energy intake). The diet includes a relative increase in the proportions of fat (≃44 %) and protein (≃43 % or ≃1·2–1·5 g/kg of ideal BM), and with a total energy intake of <800 kcal/d, depending on the amount and quality of protein preparations^(34)^.

### Ketogenic Mediterranean diet/Modified Mediterranean Ketogenic diet

The Mediterranean version of the KD has been widely studied in previous years. Basically, it is a very LCD (carbohydrate lower than 30/50 g/d) in which emphasis is placed on the intake of lean meats, fish, olive oil, walnuts and salad^(35–40)^ and, in some protocols, the addition of herbal extracts^(41–45)^.

## Food selections in ketogenic diet

Food selection is a major consideration for individuals undergoing a KD. High-carbohydrate food consumption is strictly controlled and limited during a KD^(46)^; however, it is not a ‘no carbohydrate diet.’ Meal preparation often incorporates unprocessed foods consisting primarily of cruciferous and leafy green vegetables, raw nuts and seeds, eggs, fish, unprocessed animal meats, high-fat dairy products and natural plant oils, including fats, avocados, coconuts and olives^(47–49)^. In addition to the KD foods listed in Table 2, ketogenic eating plans frequently promote meals such as omelettes, salads and animal protein such as steak, salmon or chicken with vegetables^(50,51)^. In addition, some proprietary/commercial meals are used that mimic the taste of carbohydrates but are very low in carbohydrates^(52,53)^.

**Table 2.** Frequently recommended foods in a ketogenic diet (KD)

Animal protein	Dairy products	Fats	Nuts and seeds	Fruits	Vegetables
Eggs	Cheese (especially full fat)	Olive oil	Almonds	Avocados	All green leafy
Meats	Cream Cheese	Fats	Flaxseeds	Strawberry	Carrot
Poultry	Cream	Coconut oil	Macadamia nuts	Lemon	Mushroom
Game	Butter	MCT oil/powder	Brazil nuts	Berries	Eggplant
Seafood	Full fat, no sugar yogurt	Avocado oil	Pecans	Olive	Tomato

## Mechanism of ketogenesis

Glucose is a vital fuel substrate for fat oxidation and central nervous system activity. Its role is particularly crucial in cell energy production because it is a precursor of oxaloacetate, a required substrate for the Krebs cycle^(54)^. The Krebs cycle also gives its intermediates in other biosynthetic processes. This intermediate pool replenishment process is called anaplerosis^(55)^. The endogenous production of glucose in the body, particularly in the liver, from lactate, glycerol and the amino acids alanine and glutamine is known as gluconeogenesis. When gluconeogenesis fails to keep pace with bodily needs for glucose, ketogenesis begins in earnest to provide an alternate source of energy^(56,57)^.

In humans and most other mammals, acetyl-CoA formed in the liver during the oxidation of fatty acids can either enter the Krebs cycle or undergo conversion to KB^(58)^. During a KD, the concentrations of glucose drop and the glucose reserve is not enough to guarantee oxaloacetate production for anaplerotic function. In this condition, the organism requires an alternative source of energy, which is found in the form of KB^(23,56,59)^. The three KB are acetoacetate (AcAc), beta-hydroxybutyrate (BHB) and acetone^(60)^. The production of KB occurs in the liver from two acetyl-CoA molecules through a metabolic process called ketogenesis^(61)^. When oxaloacetate is not available due to a shortage of glucose, acetyl-CoA accumulates and spontaneously diverts into the formation of AcAc, and then BHB^(56)^. Two molecules of acetyl-CoA catalysed by thiolase and produce acetoacetyl-CoA^(62–64)^. The acetoacetyl-CoA then condenses with acetyl-CoA to form beta-hydroxy-beta-methylglutaryl-CoA cleaved to free AcAc and acetyl-CoA. The AcAc is reversibly reduced by BHB dehydrogenase, a mitochondrial enzyme, to BHB. AcAc can also form acetone. In healthy people, acetone is formed in very small amounts either from AcAc, which is easily decarboxylated spontaneously or by the action of AcAc decarboxylase.

KB are then released into the bloodstream and can be absorbed by other tissues to be reconverted to acetyl-CoA and therefore provide a fuel substrate for the Krebs cycle^(65)^. This process is of importance for the brain due to its incapability to utilise directly NEFA as a source of energy. NEFA are unable to cross the blood–brain barrier. For this reason, the brain ordinarily uses glucose and, in low glucose conditions, becomes dependent upon KB^(61)^. The rapid rise of circulating KB leads to ketonaemia and ketonuria. Excretion of acetone, the volatile KB, through the lungs causes the characteristic sickly-sweet odour of ketosis^(66)^.

## Nutritional ketosis and mechanisms of ketogenic diet

Previously, interest in the KD focused on its role in epilepsy and expanded upon our knowledge of underlying biochemical mechanisms in both normal and pathologic brain function^(67,68)^. The KD acts by inducing a state of physiological ketosis, which has been linked metabolically to some anticonvulsant properties via reduced glucose, elevated fatty acid concentrations and enhanced bioenergetics reserves^(69)^. Besides, regarding its effects on brain function and anticonvulsant effects, KD affect numerous other physiological and biochemical processes. Dramatically reducing carbohydrate intake and thus decrements of insulin and leptin and increased glucagon concentrations also play a role in regulating protein and TAG balance, which results in reduced lipogenesis while increasing lipolysis^(70,71)^. Interestingly, fuel sources in a KD are fatty acids (70 % of energetic requirements from dietary fat and lipolysis of adipose tissue pools), KB (20 % of energetic requirements from lipolysis and ketogenesis adipose stores) and glucose (10 % of energy requirements from gluconeogenesis)^(72)^. Numerous factors such as BMR, BMI and BFP may be improved through ketogenesis^(23,73)^. Ketosis induced by nutritional strategy preserves concentrations of KB at a physiological status without varying the blood pH and, consequently, is considered relatively safe^(74–76)^. The body begins using primarily ketones as energy fuel after a few days or weeks from the beginning of the diet. This phenomenon is called ‘keto-adaptation’ and can vary between individuals. The mechanisms that promote keto-adaptation are still poorly understood; however, some authors have proposed the hypothesis that mitochondrial biogenesis and decrements of mitochondrial damage in oxidative tissues (such as brain and muscle) may be one of the possible mechanisms^(77,78)^. For example, studies on muscle tissue showed that a KD could contribute to mitochondrial biogenesis and reduce mitochondrial autophagy, contributing to a rich mitochondrial reservoir in the muscle tissue, enhancing exercise performance and athletic’ well-being^(79,80)^. Others believe that KB can reduce histone deacetylation, which acts as active signalling molecules and promotes important epigenetic modifications^(76,81)^.

## Side effects of ketogenic diets

KD’ serious complications appear to be rare; however, pre-existing conditions such as porphyria, pyruvate carboxylase deficiency, defects in fatty acids oxidation and mitochondrial disorders have reportedly worsened over time^(82)^. Adverse events encountered during KD can be categorised into short-term and long-term side effects.

Dehydration is typically characterised by dry mouth, headache, dizziness/orthostatic hypotension and electrolyte abnormalities (such as hyponatraemia and hypomagnesaemia), and visual disturbance is the most common short-term side effect^(83)^. Furthermore, hypoglycaemia (due to carbohydrate restriction), lethargy (due to switching from utilising carbohydrates to fat for ATP production), halitosis (caused by ketosis and increasing in acetone concentrations), gastrointestinal disturbances, involving nausea/vomiting, diarrhoea or constipation (due to gastrointestinal response to high fat intake), and hyperuricaemia are other short-term side effects of KD^(83–85)^.

Long-term side effects of KD include hypoproteinaemia (as a consequence of gluconeogenesis following carbohydrate restriction especially accomplished with low protein intake), hypocalcaemia and bone damage (probably due to low Ca intake), increasing LDL, urolithiasis (represented by chronic acidosis, dehydration and fat malabsorption), gallstones (due to rapid BM loss) and hair loss (especially when protein intake is insufficient)^(83)^.

## Effects of ketogenic diet on body mass and fat mass loss

During recent years, KD have been commonly considered a beneficial strategy to treat numerous diseases and BM and FM control. In fact, many studies suggest that they could be more efficient than low-fat diets (LFD)^(86–89)^. The efficacy of KD on BM and FM loss is related to predisposing factors, and its possible mechanisms are mainly a reduction of energy intake and appetite and an increase in daily energy expenditure.

Regarding predisposing factors, numerous findings have shown that baseline insulin dynamics or genotype patterns could play an important role in the success of a LFD *v*. a KD on BM loss^(90–94)^. For instance, individuals with greater insulin resistance might be more successful following KD due to the reduced requirement on insulin to clear a lower quantity of dietary carbohydrates delivered in the blood circulation^(90)^. Rock *et al.* showed that insulin-sensitive women lost more BM at 12 months in the LFD than the LCD group^(95)^. However, some studies did not reveal differential effects following the low fat *v*. LCD on BM loss by baseline insulin status^(96,97)^. Moreover, some studies have reported that genotype variation could predispose individuals to differentially respond to BM loss influenced by diet type^(98,99)^. In the first retrospective study, a 3-fold difference was observed following 12-month BM loss for initially overweight women who were determined to have been appropriately matched (mean BM loss of 6 kg) *v*. mismatched (mean BM loss of 2 kg) to a low-fat or LCD based on multilocus genotype patterns with SNP from three genes (PPAR Gamma, Adrenoceptor Beta 2 and Fatty Acid Binding Protein 2) relevant to fat and carbohydrate metabolism (a putative low-fat-responsive genotype and a low carbohydrate-responsive genotype, respectively). The participants with the low-fat-responsive genotype were observed to lose more BM when assigned to an LFD than those assigned to an LCD, and vice versa for those with the low-carbohydrate-responsive genotype^(99,100)^.

Adipose tissue is the main target of a BM loss programme. KD are based on the premise that reducing carbohydrate intake results in increased fat oxidation. Average interstitial glycerol concentrations (index of lipolysis) were higher following a short-term high-fat diet than an LFD based on the US Department of Agriculture food guide pyramid^(101)^. Reducing dietary fat intake in LFD can be an effective method to reduce energy intake and promote BM and FM loss compared with carbohydrate, protein and mixed meals^(102)^. In addition, in non-KD, fat intake does not immediately increase fat oxidation^(103)^. The amount of fatty acids that avoids capitation by adipose tissue appears to be small. It is insufficient to compensate for the decrease in NEFA release through insulin secretion in response to carbohydrates, usually consumed, and fats^(104)^. Conversely, KD reduce insulin concentrations, and this reduction promotes lipolysis, fat oxidation and increases energy expenditure^(105,106)^. However, the metabolic advantage and hyperinsulinaemic effects of the KD (the carbohydrate–insulin model of obesity) that claims diets rich in carbohydrates are particularly fattening due to their propensity to elevate insulin secretion, which was not evidenced in previous studies^(107,108)^. Although it is well-established that KD can be effective in FM loss, it seems that long-term (>6 months) periods may not be more effective than a well-balanced, energy-restricted diet^(109–112)^.

Previous studies have suggested that on an energy-for-energy basis, proteins are more satiating than either carbohydrates or fats^(113,114)^, and it can be suggested that the higher protein intake in KD plays a critical role in limiting food intake^(115)^. Alternatively, Westerterp-Plantenga *et al.* showed higher satiety scores with high-protein and high-carbohydrate diets (protein/carbohydrate/fat: 29/61/10) even over a 24-h period when compared with a high-fat diet (protein/carbohydrate/fat: 9/30/61), accrediting to fat content, the greater sense of hunger after a meal^(116)^. A well-designed randomised crossover study has shown that high-protein, low-carbohydrate KD reduce hunger and lower food intake significantly more than high-protein, medium-carbohydrate non-KD^(117)^, suggesting that reduced carbohydrate intake resulted in a decrease of energy intake of 0·7 MJ/d (294 kcal/d) and a corresponding effect on negative energy balance. However, another study in which carbohydrate percentage was kept at 50 %, while the protein was modified from 15 % to 30 %, demonstrated that greater protein intake could positively affect satiety, probably through a mechanism linked to leptin sensitivity in central nervous system^(118)^.

The concentrations of several hormones and nutrients influence appetite and are altered after BM loss induced by a KD^(119,120)^. Human studies have found that a higher insulinaemic response to meals may increase food intake^(121–123)^. Some studies showed that a strict LCD reduced appetite by decreasing insulin concentrations^(16,124,125)^. Moreover, other studies have shown a decrease in leptin and increased ghrelin concentrations, which are two hormones that regulate satiety; however, these effects were mitigated when BM-reduced participants were ketotic^(119,124)^. The Liver-derived fibroblast growth factor 21 is an endocrine regulator of the ketotic state and maybe another possible mechanism for appetite suppression following KD^(126)^.

Regarding animal studies, it has been previously revealed that hepatic expression and liver-derived fibroblast growth factor 21 concentrations are induced through both KD and fasting states and are quickly suppressed by refeeding^(126)^. Liver-derived fibroblast growth factor 21 also induces gluconeogenesis, fatty acid oxidation and ketogenesis, a metabolic profile characteristic of fasting^(127)^. It has also been suggested that the anorexic effects of protein may contribute to the BM loss produced by LCD^(128)^.

Furthermore, it has been proposed that limited food choices may be another cause of decreasing energy intake in KD’s followers^(129,130)^. A meta-analysis study showed a lower hunger and desire for energy intake in individuals adhering to KD^(131)^. In addition, a large number of *ad libitum* eating studies showed that KD resulted in lower energy intake^(86,89)^. However, no significant differences were noted between KD and very-low-energy diets in appetite suppression^(131,132)^. It seems that increased dietary fat oxidation and an increase in the concentration of BHB (i.e. ketosis) may contribute to the increased appetite suppression on a high-protein, LCD, and high-fat diet^(132)^. As suggested in a recent meta-analysis, it is challenging to define a ‘threshold’ of circulating ketone for appetite suppression^(131)^. However, studies have shown that BHB concentrations of 0·5 mm or even lower may be a potential threshold for appetite control, while higher concentrations (and accordingly more severe dietary carbohydrate restriction) may not be necessary to prevent an increase in appetite in response to energy restriction^(133,134)^.

It has been hypothesised that KD may reduce BM and FM by increasing daily energy expenditure^(16)^. The higher thermic effects of high-protein diets such as KD can cause increases in total daily energy expenditure^(135–137)^. Nevertheless, it has been formerly indicated that high-fat diets would generate a more metabolically effective state than glucose, and carbohydrates might produce more post-prandial thermogenesis than fats^(138)^. Indeed, per energy, carbohydrates produce about 3-fold higher thermogenesis than fats (approximately 5–10 % for carbohydrates *v*. 3 % for fat)^(139)^, while proteins have greater thermogenic effects (approximately 20–30 %). Therefore, due to significant protein intake, KD could be considered an ‘expensive’ diet and consequently increased BM loss compared with other ‘less-expensive diets^(140–142)^.

On the other hand, some authors encourage the hypothesis of a different metabolic benefit of KD on BM loss^(140)^. Glycogen store depletion may encourage the body to switch the use of the particular energy-producing process such as gluconeogenesis and ketogenesis^(15,143)^. The required energy for gluconeogenesis has been estimated at about 400–600 kcal/d^(137,141)^. Compared with an isoenergetic high-carbohydrate diet, the metabolic advantage is estimated to be approximately 200 to 300 more energy content burned^(71,144)^. Reduction in the resting RQ and, therefore, a greater percentage of fats consumed for given total energy expenditure may represent another possible mechanism of KD’s BM loss efficacy. It has been suggested that one of the main BM loss mechanisms of the KD might be attributed to an improvement in resting nutrient oxidation, and interestingly, this effect was long-lasting for at least 20 d following cessation of the KD^(145)^. Consistent with the metabolic advantages of carbohydrate-restricted diets, Ebbeling *et al.* showed a linear trend of 52 kcal/d for every 10 % decrease in the contribution of carbohydrate to total energy intake^(71)^. Compared with high-carbohydrate diets, the authors reported that the change in total energy expenditure was 91 kcal/d greater in the moderate-carbohydrate diet and 209 kcal/d greater following LCD. In this study, the carbohydrate intake was 60, 40 and 20 % of daily energy in high, moderate and LCD, protein fixed at 20 % of daily energy intake, and fat were 20, 40 and 60 %, respectively. Although Ebbeling *et al.* showed metabolic advantages of carbohydrate-restricted strategies, they did not determine total energy expenditure changes following very low-carbohydrate KD. However, Hall *et al.* did not support a large metabolic advantage following a KD^(146)^. In this study, authors investigated changes in energy expenditure, RQ and body composition in participants consuming a high-carbohydrate baseline diet for 4 weeks, followed by 4 weeks of an isoenergetic KD with clamped protein. The results showed that large isoenergetic changes in the proportion of dietary carbohydrates to fat transiently increase energy expenditure by only about 100 kcal/d after adjusting for BM and composition. The authors also mentioned that the BM and composition adjustments likely overestimated the energy expenditure changes during the KD because much of the BM loss was likely attributed to fluid loss rather than loss of metabolically active tissues (adipose tissue etc.). Another study by Hall *et al*. showed a trend for a greater degree of negative energy balance during a fat-reducing diet compared with an isoenergetic carbohydrate-reducing diet, but this was not statistically significant^(147)^. These data from different studies suggest that if there are any metabolic advantages following KD, they could be quite small. Future studies are needed to investigate the energy expenditure changes following KD and non-KD such as LFD.

Mammals have evolved to utilise carbohydrates as their primary source of metabolic fuel, extracting energy through a series of intricate biochemical pathways^(148)^. The KD mimics the metabolic state of starvation, forcing the body to utilise fat as its primary source of energy^(149)^. Many studies have shown that this kind of nutritional approach has a solid physiological and biochemical basis, inducing effective FM loss^(117,137,145,150,151)^. It has been mentioned that there is an increase in lipolysis (due to reduced insulin concentrations) and promotion of BM loss by assessment of body composition in those following a KD^(152)^. The higher amount of lipolysis may have resulted in a higher rate of FM loss following a KD. Many studies have shown that carbohydrate-restricted diets promote greater BM loss than conventional energy-restricted LFD^(89,109,130,153,154)^. However, a 36-month follow-up by Cardillo et al. showed that mean BM changes between baseline and 36 months were not different between the low-carbohydrate/high-protein and the LFD/high-carbohydrate diet group^(155)^. In non-KD conditions, it seems that individuals with obesity showed no significant differences between LFD and high-fat diets during BM loss^(10,156)^. In addition, a meta-regression of eighty-seven studies showed that LCD were associated with a greater BM loss compared with high-carbohydrate diets, which was independent of energy intake^(157)^. It seems that the BM loss observed in such diets follows a biphasic pattern due to metabolic alterations, while later BM loss is more than likely attributable to restrictive food choices. It certainly seems that initial BM loss can be attributed to diuresis; KB excretion (ketonuria) increases renal Na and hence urinary water loss^(146,158)^. In addition, glycogenolysis, a prominent feature of the early stage of a KD, is associated with concomitant water release (for every 1 g of glycogen stored, approximately 3 g of water is stored)^(159–161)^.

Based on previously mentioned potential mechanisms, it seems that initial BM loss can be attributed to dieresis. KB excretion (ketonuria) increases renal Na; hence, urinary water loss and the long-term benefits of adhering to a KD on BM loss are decreased energy intake and appetite suppression. Moreover, based on the data derived from isoenergetic studies, there are no significant metabolic advantages in following KD in increasing energy expenditure. However, some short-term isoenergetic studies reported a higher BM loss following a KD than LFD^(162–164)^, mainly because of diet-induced diuresis. The findings from isoenergetic studies underlined the ‘the calorie in, calorie out’ hypothesis, which stated that BM loss is not primarily determined by varying proportions of carbohydrate and fat in the diet but by the number of energy content ingested^(165,166)^.

Similar to BM loss, there is a body of evidence suggesting greater FM loss by adhering to a KD instead of an LFD. In addition, the findings of a well-designed randomised controlled trial found preferential FM loss in the trunk region with a KD, which was approximately 3-fold greater than an LFD^(167)^, which may have important implications for CVD treatment. Moreover, there is some evidence behind the FM-reducing effects of a KD. In general, using fat as the primary fuel source often results in greater benefits for FM loss and improved body composition^(168)^. Furthermore, KD suppress appetite and have some metabolic advantages, as previously discussed. In adults, ketones are primarily derived from long-chain fatty acids stored in adipose tissue^(169)^ controlled by insulin^(170)^. When blood glucose and insulin decrease, stimulating lipolysis allows plasma-NEFA to increase^(171)^. The increase in plasma-NEFA helps meet the need for an alternative fuel to glucose for most tissues, except the brain’s notable exception^(172)^. The increased supply of NEFA entering the liver leads to ketogenesis by condensation of two acetyl-CoA, which are present in excess due to fatty acid beta-oxidation^(173)^.

In conclusion, a KD could be beneficial in BM loss. The anti-obesity effects of KD are mainly through lowered energy intake. Moreover, controlling appetite (induced by nutritional ketosis and higher daily protein intake), restrictive food choices, increasing energy expenditure, higher lipolysis and diuresis are other possible mechanisms that help BM loss in individuals adhering to a KD. In regard to body fat, KD may be a practical dietary approach for FM loss. Short-term studies demonstrate a strong FM loss effect on KD compared with non-KD^(174,175)^. However, although long-term studies reported that adhering to a KD achieves a greater BM loss compared with those adhering to an LFD^(176,177)^, the data relating to the long-term effects of KD on FM are limited^(178)^. Most long-term studies determined the KD’ effects on body fat compared with very-low-energy KD with low-energy diets^(179,180)^. Obviously, in these studies, patients with obesity who followed very low-energy KD experienced lower body fat loss. Since very-low-energy KD consumed significantly lower amounts of energy content in these studies, the lower body fat loss in the very-low-energy KD group is related to more energy restriction, but not the benefit of KD. Alternatively, in the most long-term studies, which evaluated the long-term effects of LCD, the carbohydrate intake was higher than 50 g/d and/or 5 % of daily energy intake^(86–88,181–185)^. Therefore, it is impossible to generalise these findings to KD. However, in long-term studies that make a comparison between a KD and a LFD, Foster *et al*. did not see any benefit of following a KD after 2 years of intervention^(178)^. In other studies by Brinkworth under planned isoenergetic conditions, both dietary patterns (very-low-carbohydrate, high-saturated-fat KD and a high-carbohydrate, LFD) resulted in similar fat loss after 1 year of intervention^(112,186)^. Therefore, in an isoenergetic condition, there is no advantage in FM loss in individuals adhering to a KD compared with a LFD. Based on the available evidence regarding FM loss, although *ad libitum* short-term studies reported significantly higher body fat loss following a KD, there is not enough evidence about additional benefits of a KD compared with a LFD in long-term studies and isoenergetic conditions. However, further studies are needed to show the long-term effects of KD compared with an LFD on body fat.

## Effect of ketogenic diet on muscle mass

The main concern surrounding KD is the potential loss of muscle mass. Regarding this topic, it is worth distinguishing between fat-free mass (FFM), the portion of the body composed of muscles, bones, ligaments, tendons, internal organs, essential fat and lean mass essential fat is not included. We will refer to FFM or lean mass accurately reporting terminology in the cited study for this review.

Theoretically, some different mechanisms were claimed in which KD may preserve muscle mass following BM loss. First, it is hypothesised that elevated BHB concentrations may have played a minor role in preventing muscle mass catabolism by reducing^(187–189)^. KB appear to depress muscle protein breakdown (MPB)^(188,190)^. Previous findings have revealed that ketones, such as AcAc and its precursor BHB, may be a relevant metabolic fuel in the context of physical activity, improving athletic performance^(191)^, myocardial ATP generation^(192)^ and protective effects on muscle tissue^(193)^. Second, low blood glucose after adhering to a KD may be a potent stimulus to growth hormone (GH) secretion^(194)^. GH has a pivotal role in regulating *in vivo* protein metabolism^(195,196)^. GH enhances protein anabolism at the whole-body level, mainly by stimulating muscle protein synthesis (MPS)^(197)^. However, previous reports from animal studies have revealed that GH concentrations are normal^(198)^ or elevated^(199)^, whereas circulating insulin-like growth factor-1 (IGF-1) concentrations is reduced in rodents fed with a KD^(198,200–202)^. The IGF-1-lowering effects of KD have also been reported in human studies^(203,204)^. These findings suggest that KD might have caused GH resistance, which could have been responsible for the IGF-1 reduction. Third, in most cases, KD are relatively high in protein^(205)^ (approximately 30–35 % of daily energy intake)^(206)^. It has been recently shown that a high-protein diet could preserve muscle mass during BM and/or fat loss phase^(207–211)^. The conceivable FFM-preserving mechanism of high-protein diets can be related to dietary protein-induced alterations in protein turnover, particularly MPS, inhibiting AMP-activated protein kinase (AMPK) phosphorylation and activating mammalian target of rapamycin complex 1 signalling^(158,212–214)^. However, it seems that, besides these possible FFM-preserving mechanisms, the amount of FFM loss is slightly higher following KD compared with non-KD^(29,215,216)^.

KD is a strategy often employed by individuals who are endeavouring to lose BM rapidly. It is well established that rapid BM loss diets are not efficient at preserving FFM^(217–220)^. Unfortunately, the main contributor to BM loss can be the result of decreased muscle mass, occurring to some extent to support the burden of adipose tissue^(221)^. Following non-KD, in participants with obesity, FFM contributes approximately 20–30 % to total BM loss^(69,70,72–75,222,223)^. It seems that this amount of FFM loss is slightly higher following KD^(215,216,224)^. This catabolic effect of KD may cause an inhibiting effect on the mechanistic target of rapamycin (mTOR) signalling pathway^(225)^. By inducing a fasting-like state, KD lead to alterations in the metabolic pathways and cellular processes such as autophagy^(226)^. In an animal model, hypercorticosteronaemia and hypoinsulinaemia, along with decreased IGF-1 secretion induced by KD, resulted in muscle atrophy via autophagy, particularly in muscle tissue that can reduce MPS^(200)^. Moreover, the KD ‘mimics’ energy restriction effects on AMPK, sirtuin-1 (SIRT-1) and PPAR-*γ* coactivator 1-*α* (PGC1-*α*), which are activated through phosphorylation and are important regulators of energy metabolism^(226)^. In skeletal muscle, the activation of the AMPK/SIRT-1 pathway promotes fatty acid oxidation but consequently inhibits MPS^(227–231)^. AMPK indirectly activates SIRT-1 in skeletal muscle by increasing NAD+^(24)^. This is accomplished through the increase in mitochondrial *β*-oxidation^(228)^ and thus increased expression of nicotinamide phosphoribosyltransferase, which is the rate-limiting enzyme in NAD+ synthesis^(232)^. Simply stated, the coordinated effects of AMPK and NAD-dependent deacetylase SIRT-1 are primarily mediated by PGC1-*α*, which is activated through phosphorylation of AMPK and deacetylation of SIRT-1^(228,230,233–237)^. PGC1-*α* relocates to the nucleus, where it functions as a transcription factor. This increases the expression of genes that code for proteins involved in fatty acid transport, fat oxidation and oxidative phosphorylation. The activation by phosphorylation of PGC1-*α* may occur in several ways involving AMPK, Ca calmodulin-dependent protein kinase and p38 mitogen-activated protein kinase signalling pathways. AMPK can act in two ways: either by activating PGC1-*α* through phosphorylation or by promoting the expression of enzymes involved in skeletal muscle oxidation and metabolism^(238)^. Additionally, in participants with obesity, skeletal muscle is less oxidative and has lower AMPK activation during the fasting state^(239)^.

At the same time, AMPK activation also inhibits mTOR signalling by boosting Tuberous Sclerosis 2, an antagonist of mTOR signalling activation, which is the most critical signalling mechanism in regulating MPS^(240)^. Although there is some evidence that these changes have health benefit effects such as modulating effects on glucose homoeostasis and insulin action, KD, similar to fasting, blunts the protein kinase b (Akt)/mTOR pathway and reduces the possibility of muscle mass gains despite energy sufficiency^(239,240)^. It is well established that increasing dietary protein intake following exercise interventions, especially resistance training (RT), attenuates BM loss-induced reduction in muscle mass^(209,241,242)^. Dietary interventions that could lead to superior muscle mass retention during BM loss would be beneficial for several reasons, including maintenance of RMR^(243)^. However, most studies show that KD have no positive effect on preserving FFM than an LFD^(29)^.

In addition to the molecular pathways involved, another possible explanation is that the body recruits amino acids (through de-amination or transamination) from muscle proteins to maintain blood glucose via gluconeogenesis. Carbohydrate restriction leads to decreases in blood glucose, and it is possible that increased gluconeogenic activity could promote MPB to provide an amino acid substrate. Consequently, the primary fuel for gluconeogenesis is the amino acid pools, along with glycerol derived from TAG^(244)^. Using amino acids through gluconeogenesis can be a reason for an increase in amino acids released from muscle tissue, resulting in muscle mass decrements^(245)^. While this is known to occur during complete fasting, KD promote a pseudo-fasted state in which the oxidation of fatty acids primarily meets energy requirements due to the lack of dietary carbohydrates, but catabolism is not as pronounced as during a complete fast^(246–248)^. For instance, it has been reported that young men with obesity lost only 3 % of FFM during a 10-d hypoenergetic KD than 65 % of BM as FFM during 10-d fasting^(246)^.

Conversely, several investigations found that KD are more effective in preserving FFM compared with LCD. For instance, Young *et al*. compared three isoenergetic (1800 kcal/d) and isonitrogenous (115 g/d) dietary interventions that differed in carbohydrate content. After 9 weeks on the 30-g, 60-g and 104-g carbohydrate diets, BM loss was 16·2 kg, 12·8 kg and 11·9 kg, respectively, and fat accounted for 95 %, 84 % and 75 % of the total BM loss, respectively^(249)^. Although these results should be interpreted with caution given the low number of participants, this study strongly suggests that KD promote FM loss while preserving muscle mass compared with LCD. While it seems that KD cause more FFM loss than a high-carbohydrate diet, this finding suggests that compared with LCD, KD may be superior to preserving FFM. Moreover, data from the study by Young *et al*. provide further evidence that supports the notion that ‘a calorie is not a calorie’^(141,250,251)^.

In addition, it has been recently shown that a high-protein diet could preserve muscle mass during BM and/or FM loss phase^(207–211)^. In most cases, a KD consists of a moderate to a high amount of protein, which generally contains animal-based high-protein sources^(252)^; an important factor for dietary protein-induced alterations in protein turnover, particularly MPS, and activating mTOR signalling^(157,212–214)^. It has been mentioned that the plausible FFM-preserving mechanism of high-protein diets can be related to dietary protein-induced alterations in protein turnover, particularly MPS, inhibiting AMPK phosphorylation and activating mTOR signalling^(157,212–214)^. Nevertheless, there are a limited number of studies comparing KD with different protein intakes. However, a KD with 40 % protein maintained muscle mass in community-dwelling elite athletes^(253)^. Therefore, it seems that increasing the proportion of daily protein intake is a practical application for preserving FFM^(254)^. For example, Volek *et al*. determined the differences between energy-restricted KD (30 % protein) and LFD (20 % protein) on BM loss and body composition in overweight men and women^(167)^. Although both men and women following KD showed a greater decline in lean mass, the differences were insignificant. Therefore, a KD with correct amounts of protein could help the preservation of FFM. However, it should be considered that exceeding protein consumption could interrupt the ketogenic process.

Positive effects of carbohydrate intake on net muscle protein balance could be another possible mechanism of higher FFM loss in KD. Although it is reported that carbohydrate consumption may not significantly affect MPS^(255,256)^, some previous studies have shown its beneficial effects on net muscle protein balance by reducing MPB^(257,258)^. These positive effects of carbohydrates may be mediated by insulin^(256,259–261)^. The anti-catabolic effect of insulin acting on MPB was confirmed in a systematic review and meta-analysis of forty-four human studies, which concluded that insulin did not significantly affect MPS but had a crucial role in reducing MPB^(262)^. According to their findings, overall, insulin significantly increased net balance protein acquisition. However, it seems that the anti-catabolic effects of carbohydrates are small compared with protein or protein plus carbohydrate intake^(257,263–267)^.

Alterations in body water during KD could also cause the differences in lean mass observed^(268)^. Readings from dual-energy X-ray absorptiometry scans and biological impedance (two commonly used methods of assessing body composition) demonstrate fluctuations in body composition that occur following variations in body water content. Furthermore, these methods generally include total body water as a component of lean mass^(103,269,270)^. Therefore, the water loss that typically occurs during the initiation of carbohydrate restriction can result in an incorrect indication of functional muscle mass loss. Yancy *et al*. showed that within the first 2 weeks of a person adhering to a KD, the individual lost a greater amount of water than those who adhered to an LFD. However, after the first 2 weeks, estimations of total body water were similar between groups^(130)^. The authors also reported that FFM changes in both groups were largely explained by changes in total body water but not lean mass tissue.

A longer duration study by Brehm *et al*. showed that similar to BM and FM, lean mass decreased more in the KD group compared with the LFD group at both 3 and 6 months. These authors also mentioned that it is implausible that differences in BM between the two groups at 3 and 6 months result from extreme changes in body water in the very low-carbohydrate dieters^(109)^. Decreasing energy intake by 500 energy content daily should result in 1 pound (0·45 kg) per week^(271)^. However, KD typically produce a 2- to 3-kg BM loss in the first week; thus, at least in the early phase of KD, diet-induced diuresis plays a vital role in BM loss^(272)^.

In conclusion, BM loss following KD, like other non-KD, may result in FFM and/or muscle mass reductions. It seems there are no specific advantages for KD compared with high carbohydrate-LFD. Moreover, it seems that this amount of lean mass loss is slightly higher following KD, especially in short-term trials. Activation of AMPK and inhibition of mTOR signalling, inducing gluconeogenesis, increasing the net balance protein acquisition, and diuresis may be the possible mechanisms of lean mass loss in individuals adhering to a KD. However, increasing the portion of protein in KD may be a practical approach for preserving muscle mass following the BM loss phase. However, it should be considered that protein intake does not have to notably modify the level of glycaemia and insulinaemia with the risk to exit the status of ketosis: a sufficient level of ketonaemia is a mandatory condition for a successful KD. It seems that the short-term adverse effects of KD on FFM are because of body water reduction. However, muscle mass reduction following long-term adherence to KD may not be related to body water. Further research is needed to determine whether the effect of KD in individuals following this dietary approach. In addition, possible mechanisms underlying the effects of KD on FFM should also be examined.

## Sex-specific effects of ketogenic diets on body composition

Although there is evidence outlining the beneficial effects of KD on BM and/or fat loss, little is known about the effect of sex differences on body composition changes induced following a KD. The sex-specific impact of different dietary interventions is important because it is generally more difficult for females to lose BM^(273)^. Females are also likely to lose less BM than males during a dietary intervention^(273)^, although they are more likely to adopt and adhere to a diet initially^(274)^. Although some evidence suggests sex-specific effects of KD in animal studies^(275,276)^, findings of the sex differences in body composition changes induced by KD in humans are limited. However, like other dietary interventions, KD may be more beneficial in men than women. For example, Lyngstad *et al*. compared body composition changes following 13 weeks of KD in men and women. According to their findings, males had a greater BM (kg and %) and FM loss than females at week 9 (BM: 17 % and 20·6 kg BM loss in men compared with 15 % and 15·3 kg BM loss in women, FM: 15·5 kg FM loss in men compared with 12·2 kg FM loss in women)^(277)^. These differences were also apparent at week 13, with males achieving a greater reduction in BM, FM and FFM (from baseline) than females.

Interestingly, although it has been suggested that females are also likely to lose more FFM than males during BM loss, Lyngstad *et al*. showed that men lost more FFM at both weeks 9 (4·9 kg *v*. 3·1 kg FFM loss in men and female, respectively) and 13 (3·2 kg *v*. 1·8 kg FFM loss in men and female, respectively)^(277)^. In another study by D’Abbondanza et al., the authors reported that men seem to experience larger benefits than females in BM and FM loss after 25 d following a KD. In terms of FFM changes, no sex-specific differences were observed. In an isoenergetic study with a moderate energy restriction of about 30 % of energy, Brinkworth *et al*. compared sex-specific differences following 8 weeks of a KD^(278)^. According to the results, males had a greater BM and FM loss than females (BM: 10 kg BM loss in men compared with 7·4 kg BM loss in women, FM: 8·2 kg FM loss in men compared with 5·2 kg FM loss in women). However, FFM decreased during both interventions at a similar amount (2 kg FM loss in men compared with 2·2 kg FM loss in women), with no effect of diet or sex.

Moreover, Volek *et al*. revealed that BM, FM and trunk FM reductions were significantly greater after a KD than the LFD for men but not for women^(167)^. Although KD’ sex-specific mechanisms of action are unclear, higher basal energy expenditure because of higher FFM in men may be the main cause of these differences^(279)^. In contrast to these findings, Gu *et al*. showed similar beneficial effects of KD on body composition in both sexes^(175)^. Further studies are needed to evaluate the sex-specific effects of KD on body composition.

## Effects of ketogenic diet and exercise on body composition

It is well-documented that exercise intervention can improve body composition, including decreasing FM and/or preserving or increasing lean mass in different populations^(280–284)^. Effects of exercise on body composition are mainly accounted for by regulation of genes, hormone concentrations (e.g. testosterone, IGF-1) and metabolic pathways (especially by activating the mTOR signalling)^(285–287)^. Although professional organisations have historically focused on endurance or aerobic training-based guidelines for BM loss and maintenance^(288)^, recent guidelines and position statements targeting BM reduction and maintenance have suggested that RT may also be effective for reducing FM^(289)^. Moreover, RT results in superior improvements in muscle mass and muscular strength^(290,291)^.

Numerous studies have demonstrated various macronutrient ratios on body composition in trained populations^(8,292–294)^. Existing sports nutrition guidelines propose carbohydrate-based or periodised carbohydrate-based diets to augment muscular adaptations to exercise^(295–297)^. Carbohydrate feeding may play an important role in improving body composition and recovery in endurance and resistance-trained individuals^(298,299)^. For example, in resistance-trained individuals, carbohydrates are suggested to augment muscle development via an increased insulin response. Specifically, insulin promotes anti-catabolic effects on muscle, thereby shifting protein balance to favour anabolism^(300)^. Co-infusion of amino acids and insulin increases amino acid delivery to muscle^(301–303)^, and it may increase MPS^(262)^. Findings from a study by Bird *et al*. indicated that 12 weeks of carbohydrate plus essential amino acid ingestion enhances muscle anabolism following RT to a greater extent than either carbohydrate or essential amino acids consumed independently^(304)^. However, in the last few years, there has been a surge in popularity in low-carbohydrate and high-fat approaches such as KD due to its purported beneficial effects on body composition^(29,238)^. Like untrained individuals, a KD may be an effective BM and FM loss strategy in athletes^(305)^. Mainly, in trained individuals, anti-obesity benefits of KD were shown in *ad libitum* studies^(306,307)^. The BM and/or FM loss may likely be explained by a resultant energy deficit created by the KD, as enhanced feelings of satiety and a reduction in overall food intake^(306)^. However, some evidence suggests that following a KD combined with exercise resulted in more fat oxidation and more ATP production from fat^(308,309)^. These findings underline the efficacy of KD on mitochondrial function and efficiency towards fat oxidation in athletes. However, there are still some concerns about FFM decrement in athletes performing high-intensity exercises^(310,311)^. In regard to the effects of a combination of exercise with a KD on adiposity, studies showed more efficacy of KD in BM and FM loss, especially in *ad libitum* conditions^(312–315)^.

The KB, BHB and AcAc are optimal substrates for muscle tissue and are rapidly oxidised. Unlike severe energy restrictions, KD provide adequate amounts of energy and protein to athletes. Therefore, KD avoid protein deficiency but induce a ‘fasting-like’ state, leading to alterations in the metabolic pathways^(238,253)^. Although both fasting and KD result in glycogen depletion and increased serum FFA, physiological adaptations following a KD are different from fasting. Losses of the magnitude encountered in fasting cannot be accounted for by adipose tissue breakdown alone and more likely represent significant lean tissue catabolism^(246)^. Since KB plays an essential role in regulating muscle substrate utilisation, these differences may cause differences in KB concentrations^(316–318)^. KB exert a restraining effect on MPB^(205)^. Thomsen *et al*. reported that BHB has potent anti-catabolic effects in muscle at the whole-body level; in muscle, reduction of MPB overrides inhibition of MPS^(188)^. Besides the dietary interventions, prolonged physical exercise performed in a fasted state also stimulates ketogenesis and results in post-exercise hyperketonaemia^(319–321)^. For example, KB concentrations can reach about 0·5–1·0 mmol/l in response to 2 h of exercise performed in an overnight fasted state and subsequently increase to about 1–4 mmol/l during early post-exercise recovery ^(321–323)^. The extent of exercise-induced hyperketonaemia during and after exercise is influenced by the intensity and volume of the exercise performed, as well as nutritional status^(319,320)^. Alternative fuelling strategies, based on adaptation to a KD, increase fat oxidation during exercise and might help spare the body’s limited glycogen stores^(324)^. In addition, KD have been used to increase fat oxidation during exercise. This also increases the production of KB, which may provide an additional energy substrate for the brain and muscle tissue^(325)^.

Moreover, higher quality and quantity of protein stimulated MPS^(326–329)^. It is well established that muscle mass gains depend highly on a net balance between MPS and MPB^(330)^. Therefore, besides the similarities between KD and fasting, a KD could positively affect muscle mass by decreasing MPB while stimulating MPS to a greater extent than fasting. However, it seems that KD are not substituted for a high-carbohydrate diet regarding preserving muscle mass.

In summary, KD can be a practical approach for BM and FM loss in both resistance and endurance-trained individuals. However, its effects on muscle mass depending on the type and intensity of training employed. Later, in this paper, we will enlarge on body composition changes in RT and endurance training (ET) athletes adhered to KD.

## Resistance training

KD combined with RT interventions may increase the rate of FM loss in athletes, but compared with non-KD, it is not an appropriate dietary approach for increasing muscle mass. While KD may be helpful in endurance performance^(191,331)^ by increasing fat oxidation capacity^(309,332)^ (especially in long-distance events lasting from 2 to 5 h), it is an oxymoron when athletes seek to boost muscle hypertrophy^(238)^. Previous animal studies suggested that KD might impair the balance between anabolic and catabolic pathways within skeletal muscle. For instance, Kennedy *et al*. reported that mice fed with a low-energy KD (79 % of fat, 10 % of protein) over 9 weeks exhibited 17 % lower absolute lean mass compared with mice fed a standard chow diet (6 % of fat, 24 % of protein)^(333)^. They also showed that KD feeding is associated with a two-fold increase in AMPK in the liver and more than a three-fold increase in the soleus muscle.

Moreover, Frommelt *et al*. reported that two KD consisting of 75 % fat, 10 % protein, 65 % of fat, 20 % of protein, reduced whole-body nitrogen balance and carcass protein content in rats compared with those fed a standard chow diet (5 % of fat and 21 % of protein) after 4 weeks^(334)^. Furthermore, it has been reported that the KD inhibits the mTOR signalling pathway by reducing the expression of Ribosomal protein S6 kinase beta-1 and Akt^(225)^. These findings have led others to contend that increased KD-induced skeletal muscle AMPK activation may blunt anabolic mTOR signalling despite energy sufficiency^(238)^. Indeed, this hypothesis is supported by several human studies that have reported that chronic KD result in attenuated muscle mass. For example, Volek *et al*. reported that despite a KD significantly reducing whole-body and abdominal fat over 12 weeks, lean mass also declined by 3·4 kg *v*. 1·0 kg in participants who were placed on LFD^(335)^. Noakes *et al*. also showed that a KD reduced lean mass by 2·6 kg over 12 weeks^(215)^. However, it should be noted that equivocal reports suggested that KD do not affect muscle mass^(253,336,337)^. It should be mentioned that higher BM decrements can result in higher FFM loss, and therefore, higher FFM loss may be the result of more BM loss during KD. In this situation, FFM percentage changes can be a more reliable index for the FFM-preserving effects of KD. Therefore, future studies should focus more on FFM percentage changes to evaluate KD’ effects on lean mass changes.

While it has been reported that KD result in a decrease in lean mass, there is limited evidence to suggest that a KD combined with RT may be beneficial for attenuating the decrease in lean mass. For instance, Jabekk *et al*. reported that while RT on a regular diet may increase lean mass without significantly affecting FM, RT combined with a KD may reduce FM without negatively affecting lean mass^(312)^. It has been revealed that adopting a KD with RT causes marked reductions in whole-body adiposity while not impacting lean mass^(338)^. In contrast, most studies reported a significant decrease in FFM following a KD with RT. In a crossover study, the KD (≤50 g or ≤10 % daily intake of carbohydrates) phase resulted in significantly lower BM (3·26 kg, *P* = 0·038) and lean mass (2·26 kg, *P* = 0·016) compared with the *ad libitum* usual diet (>250 g daily intake of carbohydrate)^(14)^. In addition, results from a study by Wood *et al*. indicated that a KD without exercise led to less FFM loss than an LFD and similar losses compared with an LFD combined with RT^(339)^. More recently, Vargas-Molina *et al*. found that in an *ad libitum* condition, a KD helped decrease more FM compared with a non-KD after 8 weeks of RT in trained women (–1·1 *v*. 0·3 kg). However, absolute changes were more favoured for non-KD (–0·7 *v*. 0·7 kg)^(340)^. Moreover, in another *ad libitum* study using US military personnel, KD combined with RT showed a remarkable BM loss compared with a normal mixed diet (–7·7 kg *v*. 0·1 kg). FM and BFP decreased in KD compared with non-KD (–5·9 kg *v*. –0·6 kg and -5·1 % *v*. –0·7 %, respectively). However, lean mass decreased in KD, while non-KD participants gained weight (–1·4 *v*. 0·8 kg)^(310)^. One possible reason that KD failed to adopt during RT is that during high-intensity exercise, the rate of ATP breakdown is too high to be matched by the rate of ATP production from FFA^(341)^. This phenomenon limits the use of fat loading in sport disciplines that require high-intensity efforts from the athletes. High-intensity exercise also suppresses lipolysis, thereby reducing the availability of fatty acids to the muscle^(342)^. An increased rate of glycolysis and lactate production during exercise also hinders fat oxidation by reducing the entry of long-chain fatty acids into the mitochondria^(343)^. On the other hand, Wilson *et al*.’s study is the only study that reported an increase in FFM after 10 weeks of KD and 2 weeks of carbohydrate reintroduction in resistance-trained males^(344)^. However, it seems that muscle mass increments in the Wilson *et al*. study were because of a 2-week carbohydrate loading, which strongly suppressed the Tuberous Sclerosis 2 protein as an antagonistic of mTOR signalling activation. It is important to note that the evaluation of FFM by Dual-energy X-ray absorptiometry includes intracellular water, which is stored in concert with muscle glycogen in a about 3:1 ratio^(159)^. Thus, another reason for increasing FFM following 2 weeks of carbohydrate refeed to the 10 weeks of KD in the study by Wilson *et al*. maybe because of increasing intracellular water which can positively influence final FFM results. Almost all of the research reported a decrease or no significant changes in FFM following a KD combined with RT. It seems that increasing protein intake preserves lean mass in resistance-trained individuals adhering to KD. Studies that reported similar (non-significant) changes in lean mass, consumed higher protein intakes in KD group (≈ 17–58 % or 18–118 g more protein intake in KD group)^(253,312,313,315,339,345)^. However, in the study by Vargas-Molina *et al*., higher protein intake (115 *v*. 97 g in KD and non-KD group, respectively) in KD could not help muscle mass preservation and there was a significant lean mass loss following KD^(340)^. In another study, Paoli *et al*. reported that KD may be used with the caution during body building preparation because it can blunt hypertrophic responses^(346)^. Recently, Vidic *et al*. compared the effects of two isoenergetic hypoenergetic ketogenic hyper-ketonaemic and non-ketogenic low-carbohydrate high-fat high cholesterol diets on body composition in strength-trained middle-aged men^(347)^. Based on their findings, these two diets have a similar impact on body composition. A recent meta-analysis of thirteen randomised controlled trial by Ashtary-Larky *et al*. showed that a combination of RT with KD was associated with declines in all body composition indices, including BM, BMI, FM, BFP and FFM^(108)^. Based on the results derived from this meta-analysis, although KD resulted in more BM and FM loss, significant changes in these two indices occurred only in *ad libitum* studies but not in isoenergetic studies. Although all included studies in the analysis lasted <3 months, the pooled results demonstrated that KD interventions resulted in 1·26 kg of FFM loss. Surprisingly, the amount of BM and FM loss was 3·67 and 2·21, respectively. These findings suggested that one-third (34 %) of BM loss in individuals performing RT may be from FFM.

In conclusion, it seems that KD may be a practical dietary approach for reducing BM and FM. In *ad libitum* studies, KD resulted in more BM and FM loss in resistance-trained individuals^(312,313)^. However, these advantages did not report in non-*ad libitum* studies (same energy restriction in both KD and non-KD groups)^(339,345)^. Moreover, there are some concerns about FFM decreasing in RT athletes who adhered to a KD in both *ad libitum* and non-*ad libitum* conditions. KD-induced skeletal muscle AMPK activation, which blunt anabolic mTOR signalling, may be a possible mechanism of lean mass loss in KD. Higher protein intakes may be beneficial to lean mass preservations in resistance-trained individuals following a KD. Further longer-term research is needed to determine the effects of KD on resistance-trained individuals.

## Endurance training

Under usual dietary conditions, athletes utilise carbohydrates as their predominant fuel source following high-volume ET^(296)^. However, it is well established that ET can increase lipolysis and help decrease FM during the BM loss phase^(348,349)^. Since the body can metabolise fat more efficiently during ET^(350)^, KD could efficiently prepare carbohydrates and promote fat oxidation^(351)^. There is robust evidence that substantial increases in fat oxidation occur, even in elite endurance athletes, within 3–4 weeks and possibly 5–10 d of adherence to a KD^(352–355)^. Previous studies involving KD have reported increases in intramuscular TAG^(356)^, hormone-sensitive lipase^(357)^, expression of fatty acid translocase FAT/CD36 protein^(358)^ and carnitine palmitoyltransferase^(359)^. Collectively, these changes suggest increases in fat availability, mobilisation and transport activities within the complex regulation of fat utilisation by muscle tissue^(357,358,360)^. Even short-term interventions have shown a reduction in respiratory exchange ratio during exercise, and it generally indicates enhanced fat oxidation^(361)^. A reduced respiratory exchange ratio has been considered a metabolic benefit of LCD^(361,362)^. However, compared with long-term studies, short-term investigations show less substantial effects on body composition, likely due to the absence of keto-adaptation^(363)^.

In a prospective, randomised, 2-week pilot study, compared with non-KD, adhering to a KD combined with ET failed to show significant improvements in body composition^(361)^. In an isoenergetic study with a moderate energy restriction of about 30 % of energy, Brinkworth *et al*. reported a slightly higher but significant BM loss in the KD group compared with a high-carbohydrate group (–8·1 and –6·7 kg, respectively) for 8 weeks^(278)^. Authors also reported similar BM loss in both diet groups for women but greater BM loss in KD than in high-carbohydrate groups for men. Similarly, there was a greater reduction in FM in men consuming the KD than the high-carbohydrate diet, but similar reductions for both diet groups in women. Finally, FFM decreased during both interventions at a similar amount, with no effect of diet or sex. In another study by Burke *et al*., BM decreased over the 3 weeks of intensified training and a mild energy deficit, with losses being greater in the KD group than the high-carbohydrate diet group^(353)^. Compared with a high-carbohydrate diet, the authors also reported that the KD was associated with the highest rates of whole-body fat oxidation ever reported across exercise of varying speeds and intensities. There is evidence that those who adhered to a KD comfortably exceeded the time frame shown to produce robust cellular adaptations to ‘retool’ the muscle to increase its capacity for fat oxidation^(314)^. Dostal *et al*. showed that 12 weeks of a KD resulted in more BM, FM and BFP decrements without any significant changes in FFM in recreationally trained individuals performing interval training and home-based and endurance-type (e.g., running, cycling, sports games) exercises^(315)^. In an *ad libitum* study by McSwiney *et al*., 12 weeks of KD showed a significantly greater decrease in BM (–0·8 *v*. –5·9 kg) and BFP (–0·7 %, *v*. −5·2 %) without any changes in lean mass (+0·1 *v*. +0·3 kg) compared with a non-KD in endurance-trained men^(314)^. A single-arm, before-and-after comparison study consisting of a 6-week KD, Urbain *et al*. revealed that a combination of ET with KD was associated with declines in all body composition indices, including BM, FM and FFM in healthy adults participating in aerobic exercises^(203)^.

However, because of the absence of a control group, these findings should be interpreted with caution. Furthermore, McSwiney *et al*. investigated the effects on substrate utilisation during incremental exercise and changes in body composition in response to 7 d *ad libitum* consumption of a KD by athletes in endurance sports^(364)^. Their finding suggested higher fat oxidation, 76 % of BM loss was from FFM decrement (–1·82 kg FFM and −2·4 kg BM-loss). However, a high FFM loss in this short-term study may be attributed to diet-induced diuresis following keto-adaptation. The body can use more fat as fuel while freeing itself from degrading muscle and liver glycogen at high rates^(365)^. In an animal study, Ma *et al*. evaluated the effects of an 8-week intervention of a KD and running on a treadmill using mice^(366)^. They found that the KD may potentially prevent muscle damage by altering the IL-6 secretion. These results suggested that a long-term KD, which warrants keto-adaptation, could be a valuable aid to endurance athletes to improve body composition by decreasing BM and body fat while possibly preserving lean mass.

It seems that the beneficial effects of KD on body composition and endurance performance in endurance-trained individuals are due to greater fat oxidation during exercise^(367–370)^. The appeal of KD for endurance athletes is likely due to the shift in fuel utilisation, from a carbohydrate-based model to one that utilises fat primarily, of which stores are virtually unlimited compared with carbohydrates (i.e. muscle glycogen)^(306)^. This metabolic shift was observed after a period of KD adhering almost named ‘fat-adapted,’ which has been well-documented in studies since the 1980s^(371)^. These adaptations may be the reason for the advantageous effects of KD on FM in endurance-trained athletes^(112)^. High-fat KD may require a significant amount of time for adaption in endurance-trained individuals^(372)^. It is common for individuals to report fatigue and energy deficiency in the first few weeks after adopting a KD^(373)^. Volek *et al*. have indicated that several months may be necessary for adaptation, fatigue symptoms to subside and adjustments in glycogen homoeostasis^(363)^. These could be potential mechanisms for longer-term studies that showed improvements in body composition and endurance performance in endurance-trained individuals.

During exercise, fat is recruited in the form of FFA (and albumin-bound FA), as very-LDL-TAG, and from muscle tissue as TAG (either from intra- or extracellular stores)^(374)^. Seven days following the start of a KD combined with ET, TAG-derived fatty acid oxidation (very-LDL or intramuscular TAG) plays a role in increasing fat oxidation and plasma-derived fatty acids remain the major source for fat oxidation^(375)^. After a 7-week adaptation to the diet and training (1 h of exercise at 50 % of maximal power output), increases in fat oxidation were derived from increased utilisation of very-LDL-TAG, plasma fatty acids^(369)^. In addition, it has been shown that high-fat diet-induced increases in muscle lipoprotein lipase activity^(376)^. Accordingly, it could be suggested that, during exercise, fat recruited from both plasma NEFA and plasma very-LDL-TAG is responsible for the increased fat oxidation after long-term high-fat diet adaptation. Intriguingly, muscle TAG utilisation is not increased after a high-fat diet considering that high dietary fat content would lead to increased muscle TAG storage, and vice versa a low dietary fat content results in decreased muscle TAG storage^(377,378)^.

Interestingly, it seems that muscle glycogen is not different following KD and high-carbohydrate diets. Volek *et al*. compared the metabolic adaptations in elite ultra-marathoners and ironman distance triathletes following a 20-month KD and high-carbohydrate diet^(363)^. They showed that muscle glycogen was significantly decreased by 62 % immediately post-exercise (a 180 min submaximal run at 64 % VO_2_max on a treadmill) and 38 % at 2 h post-exercise in the high-carbohydrate diet group, while in the KD group, muscle glycogen was decreased by 66 % immediately post-exercise and 34 % at 2 h post-exercise. In contrast, two-fold higher rates of peak fat oxidation were detected during graded exercise in the KD group, greater capacity to oxidise fat at higher exercise intensities and two-fold higher rates of fat oxidation during sustained submaximal running^(363)^. Besides, the effects of KD combined with ET on body composition and the impact of carbohydrate loading are unclear. Only one study investigated 7-d carbohydrate loading following KD and increased BM, FFM and FM, which may be related to the increased blood concentration of insulin and glucose responsible for increasing the rate of lipogenesis, as shown through increased BM and FM^(379)^. It seems that increments in FFM after the 7-d carbohydrate loading procedure were most likely due to the increased carbohydrate intake and greater synthesis and storage of muscle glycogen^(380)^.

Regarding high-intensity interval training (HIIT), there is limited data about KD’ effects in individuals performing HIIT. In an *ad libitum* study, Cipryan *et al*. evaluated the effects of altering from a habitual mixed Western-based diet to a KD over a 4-week time course during HIIT^(381)^. BM (–4·7 *v*. −0·8 kg) and BFP (–3·2 *v*. −1·1 %) decreased more in the KD trial. Moreover, in a crossover study, Gyorkos *et al*. determined the influences of a KD with and without HIIT exercise in participants with the metabolic syndrome^(382)^. Their findings showed that KD with and without HIIT significantly improved body composition by decreasing BM, BFP and waist circumference compared with baseline. However, the addition of HIIT to KD improved body composition (BM, BFP and waist circumference) more than following a diet alone. To the best of our knowledge, there is no study to determine the effects of a KD combined with HIIT on lean mass. Since the impact of a KD combined with HIIT has not been adequately studied, further studies are needed.

Studies suggested that KD are a practical dietary approach for improving body composition in ET athletes by decreasing BM and FM while probably preserving FFM. According to current evidence, it seems that the FFM-preserving effects of KD are more efficient in endurance-trained than resistance-trained individuals. It also appears that the beneficial effects of KD on body composition in endurance-trained individuals are due to shifting fuel utilisation toward greater fat oxidation during exercise, which occurred after adaptation to a KD. These findings underlined better adaptation of KD in endurance-trained individuals.

## Conclusions

A KD may help improve body composition by decreasing BM and body fat by controlling hunger and improving fat oxidation in both individuals with obesity in athletic populations. Regarding BM and body fat loss effects of KD, KD do not have any superior benefit than non-KD in individuals with obesity and athletes in an isoenergetic situation. In sedentary individuals with obesity, it seems that FFM changes appear to be as great, if not greater, than decreases following an LFD. However, there are some concerns regarding the FFM decrement in individuals following KD, especially in resistance-trained athletes. Moreover, the FFM-preserving effects of KD are more efficient in athletes performing ET compared with resistance-trained individuals. Future well-controlled research (isoenergetic and iso-protein) should be conducted in participants of different ages and various training experiences (e.g. novice, trained or elite).
